# Bladder wall and surrounding tissue necrosis following bilateral superselective embolization of internal iliac artery branches due to uncontrollable haematuria related to bladder tumor: case report

**DOI:** 10.1186/s42155-018-0043-z

**Published:** 2018-12-06

**Authors:** Andrey Tarkhanov, Gabriel Bartal, Sergey Druzhin, Rafael Shakhbazyan, Evgenii Grebenev, Maxim Kartashov

**Affiliations:** 1 Sverdlovsk Regional Oncology Center - Interventional Radiology, Yekaterinburg, Russia; 2 Meir MC (Medical Center), Radiology, Kfar-Saba, IL Israel

**Keywords:** Bladder tumor embolization, Lower urinary tract bleeding, Bladder malignancies, Embolization particles

## Abstract

**Background:**

Case of urinary bladder wall and surrounding tissue necrosis following bilateral superselective embolization of internal iliac artery branches due to unmanageable haematuria associated with aggressive bladder tumor.

**Case:**

We achieved the bleeding control, but patient demonstrated severe postembolization syndrome at follow-up (low abdominal pain, arterial hypertension, hyperthermia). Severe bladder tissue and surrounding neoplastic tissue necrosis developed several days after procedure. Patient died from multiple organ dysfunction syndrome due to longstanding peritonitis.

**Conclusions:**

Tumor ischemia and bladder wall and surrounding tissue necrosis, are possible serious complications ofembolization using calibrated microspheres. These complications can be very dangerous, and even fatal.

## Background

Embolization of bleeding due to locally advanced bladder tumors is one of the most effective and minimally invasive methods, especially in cases of non-operative bleeding that doesn‘t react to conservative treatment. Severe complications are rare, but if they occur, patient’s well-being would be at risk (Korkmaz et al., 2016; Greenstein et al., 1983). Complications can manifest as fever, pelvic pain, nausea (all of this is associated with postembolization syndrome and can be managed with NSAIDs). Minor complications can occur on first postprocedural days. It can be infection, urinary incontinence, retention of urine, prostatitis, soft tissue and penile ischemia (Greenstein et al., 1983). All of the conditions should be managed conservatively.Recently several case reports with positive outcome have been published (Vikash Prasad, 2009; Korkmaz et al., 2016; Pereira et al., 2000; Pereira & Phan, 2004; Carmignani et al., 1980; El-Assmy & Mohsen, 2007). In this communication we describe case of tumor and surrounding bladder tissue necrosis following bladder arteries embolization due to macrohaematuria.

## Case presentation

A 58-year-old male arrived at policlinic. He complained on dysuria, ischuria for several months and macrohaematuria. He did not have physical symptoms of acute blood loss. Serum Creatinine level was normal (68 mg/dL). Ultrasound examination of kidneys and urinary tract revealed solid bladder mass of about 55 mm in diameter without signs of metastatic spread. Patient had no significant anemia (HGB 117 g/l), but HGB level decreased from 143 g/l in two days.

Before hospitalization additional imaging was performed:

- MRI: bladder tumor 63 mm with invasion of the right ureter with right ureterohydronephrosis.
- On nephroscintigraphy: nonfunctioning right kidney.
- Irrigation through the three-way catheter was ineffective.
- Conservative treatment of haematuria (Транексам® (Tranexam), Obninsk, Russian Federation, tablets 500 mg 3 times per day) was ineffective.
- Cystoscopy was non-informative due to profuse haematuria, with multiple blood clots in the bladder.

After following discussion with referring physician it was decided to perform selective embolization of bladder tumor due to potential life-threatening blood loss. Pre-operative CT-Angiography showed significant neovasculaturization of the tumor without obvious source of active bleeding.

**Fig. 1:**
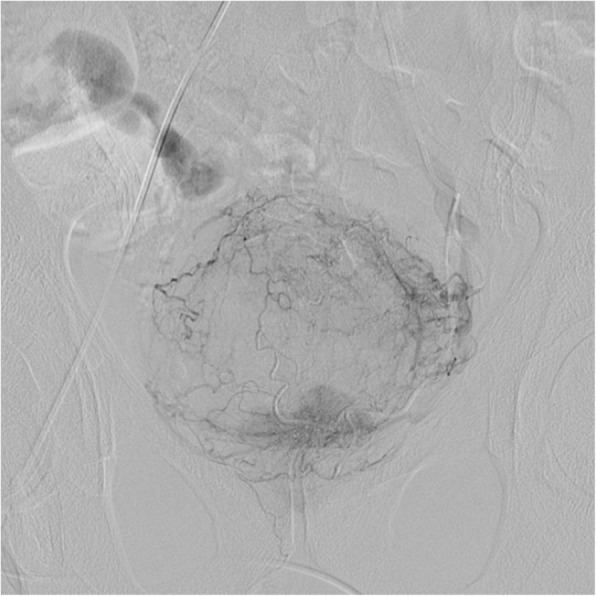
Neovascularization in projection of tumor bed

**Fig. 2:**
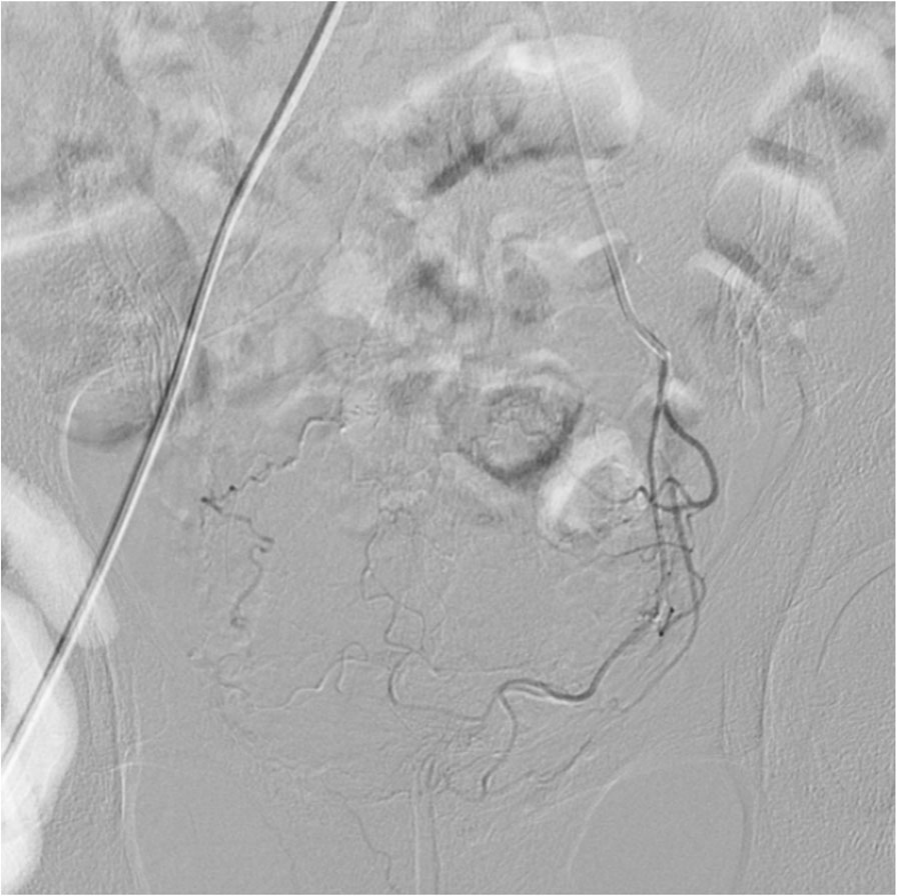
Finishing embolization on the left side

**Fig. 3:**
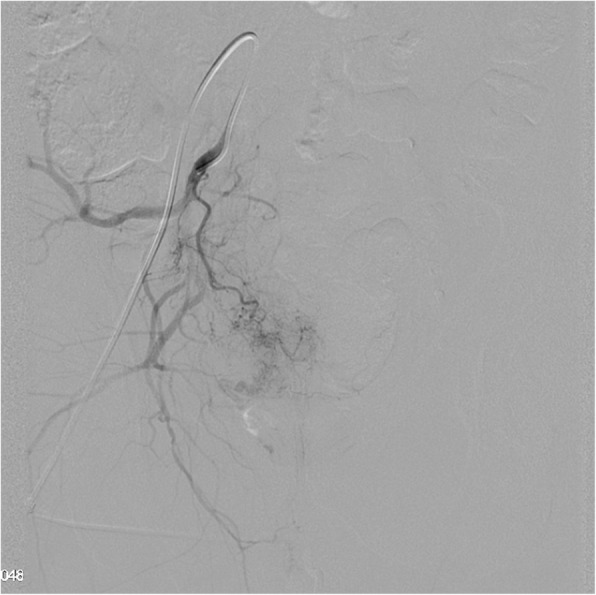
Finishing embolization on the right side

**Fig. 4:**
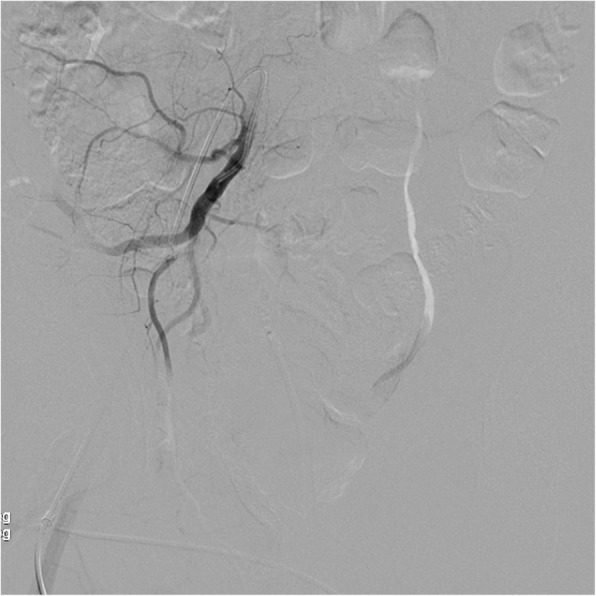
Right side after finishing the procedure

Patient underwent an embolization procedure: Right Common Femoral artery access (Vascular sheath Prelude 5F, Merit Medical, USA., catheter Cobra C2 5F, Merit Medical, U.S.A.), bilateral superselective embolization of anterior division branches of Internal Iliac Arteries using microcatheter 2,4F (Progreat-α micro catheter, Terumo Corporation, Japan) and Avigo hydrophilic guidewire, Medtronic, USA). Superior and inferiorvesical arteries on both sides (Figs. 1 and 2), left prostatic artery and left obturator arteries were embolized using 500 μm Embozene particles (Boston Scientific Corporation, USA) 1 vial per procedure (diluted with contrast media and saline in amount to stable suspension) to achieve subtotal stasis (near absence of contrast enhancement of distal parts of above-mentioned arteries), subtotal embolization of both anterior portions of Internal Iliac Arteries (notable ceasing of contrast flow from arising point) upon exit using gelfoam (Pfizer, USA) and Embozene suspension (self-made combination of Gelfoam particles about 20% of 10 ml syringe volume, mixed with Embozene spheres about 10% of syringe volume with contrast media and saline) (Figs. 3 and 4). Such comprehensive embolization was performed as our dedicated medical facility with determined equipment is exclusive in terms of treating oncology patients and this particular patient can’t be brought here in time if bleeding reoccure at home.

**Fig. 5:**
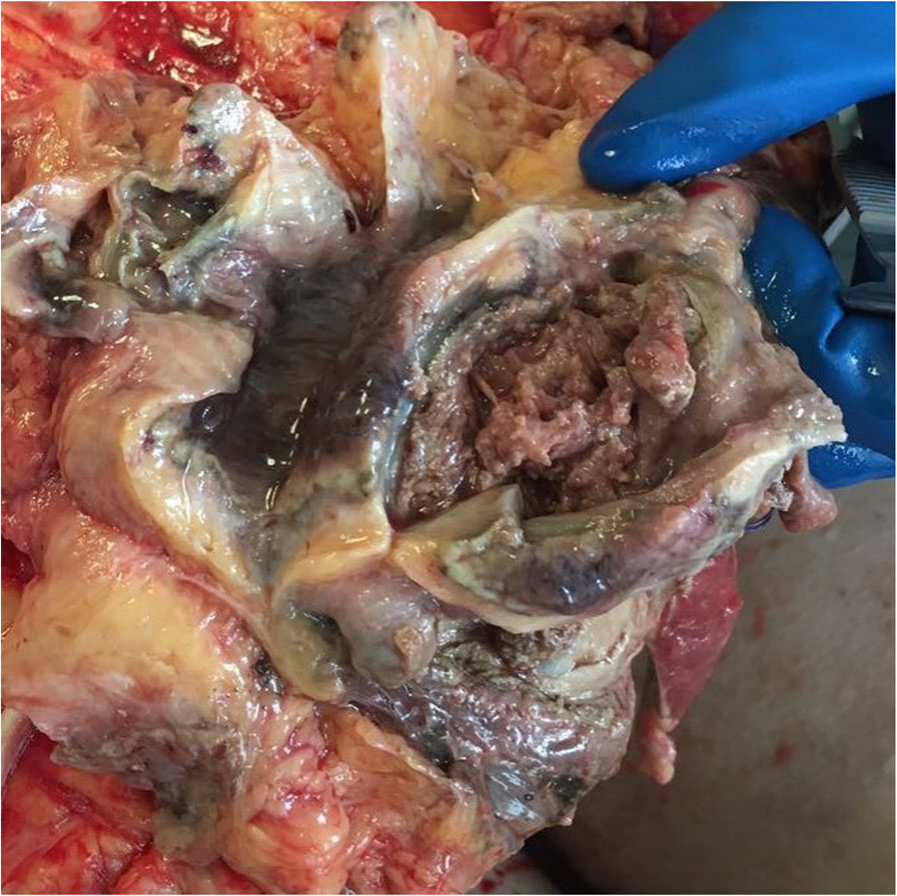
Totally necrotized tumor and underlying bladder wall

**Fig. 6:**
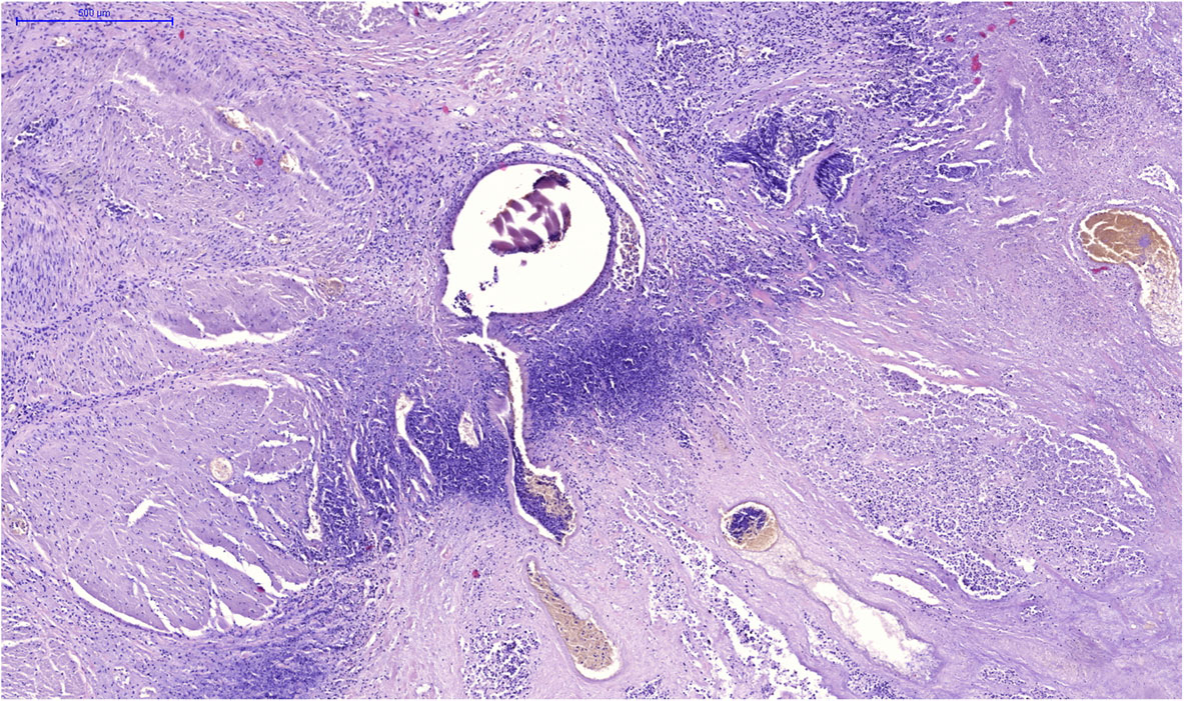
Microscopic view (normal muscle tissue, demarcation zone, tumor tissue and microembolus in vessel)

The procedures were performed under local anesthesia using Lidocain 1%. Intravenous Fentanyl 0,2 mg (Fentanyl, Moscow endocrine factory, Moscow, Russia) was injected before embolization for analgesia during the procedure and programmed IV morphine pump connected following the procedure. Patient suffered from severe pelvic pain for 4 h after the procedure and mild pain in pelvic area for 2 days. Haematuria ceased on day 3 after the procedure. For planning further treatment transurethral biopsy of the mass was performed, necrotic villous papillary tumor in lower semicircle of the bladder was visualized. Two days later urologist diagnosed peritonitis. On Ultrasound examination massive amount of free hyperechoic liquid, without gas was reported. Urgent exploratory laparotomy shown 3 to 4 l of free muddy smelly fluid in the abdominal cavity and total necrosis of the bladder wall. Sanation of abdominal cavity, necrectomy, cystostomy, and bilateral nephrostomy were performed. Patient deceased from multiple organ failure on day eight despite symptomatic therapy in intensive care unit. An autopsy report described total necrosis of tumor with necrosis of underlying bladder wall and multiple organ failure due to sepsis (Figs. 5 and 6).

## Conclusion

Chronic bleeding has been described in cases of most significant problems of oncourology. Oncologic patient can face a permanent cascade of disbalance between pathways of coagulation (Fricke et al., 2017; Salignac et al., 2002; Kennedy et al., 2016; Zhu et al., 2016). These patientsare in slightly greater risk of bleeding without any surgical procedure and greater risk of bleeding in post-operative period. The most common cause of such complication could be a diffuse bleeding from traumatized tumor tissue surface after cystoscopic punch-biopsy. Conservative methods of bleeding control (Kolev & Longstaff, 2016) (fibrinolysis inhibitors) will be ineffective in most cases. It’s a complex situation, because not each all patient from this cohort can survive cystostomy due to poor performance status. Recently, superselective embolization became the method of choice to control such bleeding. Besides, physicians choose embolization among other methods of treatment for acute bleeding: combined trauma with pelvic fractures, uncontrollable bleedings after gynecological surgical operations and complications of radiotherapy (may be chronic as well) (Agolini et al., 1997; Matityahu et al., 2013; Ali et al., 2014; Sieber, 1994; Samuel Washington & Benjamin, 2016; El-Shalakany, 2003). Usually, sequence of actions comprises bilateral diagnostic angiography of internal iliac arteries in order to determine a source of bleeding (arterial supply of bladder tumors can originate from superior and/or inferior vesical arteries, prostatic arteries, not infrequently it may be branches of internal pudendal artery, obturator arteries (Vikash Prasad, 2009)) followed by superselective embolization using various embolic agents. The most frequently-used agents are embolic spheres of different sizes, that can provide permanent tumor vessels occlusion (choosing spheres diameter depends on expected vessel penetration), but IRs also can use polymorphic PVA agents (also permanent occlusion), gelfoam particles (temporary occlusion) or combinations of those. Described method are effective alternative to surgery. Embolization can be performed even in the in patients in shock and with high procedure success. The patient does not require special preparations. Despite obvious advantages and minimally invasive character of the procedure, in some cases embolization can lead to serious complications. In retrospective study of Matityahu et al. (Matityahu et al., 2013) authors report 11% chance of complications, including one case of bladder necrosis from cohort of 98 patients. Serious complications were reported in all patients who underwent bilateral embolization. A major complication as bladder necrosis is described within the first 4–5 weeks following the embolization (Ali et al., 2014; Sieber, 1994), however, we have found data of bladder necrosis in earlier post-operative period (two weeks after intervention) (Sieber, 1994; Samuel Washington & Benjamin, 2016).

In our previous experience with over 40 embolizations of bleeding Bladder tumors we used spherical calibrated BeadBlock (BTG) and EmboGold by Merit Medical particles we did not observe such complications with similar aggressive character of performed procedures.

In our patient bladder necrosis occurred within 10 postembolization days. Factors that may contribute in developing of such life-threatening condition: tissue hypoperfusion and potential non-target embolization of non-involved in tumor supply bladder arteries, lack of good collateral flow due to patient abnormality such as atherosclerosis, hypovolemia due to developed sepsis. We associate this phenomenon with use of strictly-calibrated microspheres with high concentration of particles per unit of volume (in comparison of non-strictly calibrated PVA particles or gelfoam). Strictly calibrated embolic agent is expected to have greater ability to penetrate the arteries with an exceptional dense filling capabilities, which can despite relatively large size of each sphere (500 μm) lead to complete tumor and some bladder wall ischemia.It seems that strictly-calibrated microspheres do provide a desired haemostatic effect, but we have to carefully chooseappropriate size of embolic agent according with vessel size of the target organ.

Methods of embolization for bleeding control are firmly established in daily practice. Indisputable advantages of embolization therapy provide an opportunity to use it for patients with poor performance status, who can’t survive surgery or have contraindications for general anesthesia. Yet, even minimally-invasive treatment with overly aggressive approach can lead to life-threatening conditions. Such potential complication has to be discussed with the patient prior to the procedure and signing the informed consent. Each individual case requires a correct estimate of tumor blood supply and collateral flow followed by subtotal embolization of vessels feeding the tumor. Based on described method of bladder tumor embolization using strictly-calibrated microspheres (EmboZene) we recommend to avoid use of particles sized 500μmor less, as it can lead to total necrosis of the tumor and surrounding bladder tissue. In situations with less accessibility of catlabs for population from very remote locations, the goal is to find balance between significantly diminish the flow in tumor vessels to achieve stable hemostasis within the limits of single procedure, but not complete cease of blood flow in the tumor bed in case of compromised collateral flow.

## Abbreviations

CT-Angiography: Computedtomographyangiography
HGB: Hemoglobin
IR: interventional radiology
IV: intravenous
MRI: Magneticresonanceimaging
NSAIDs: Nonsteroidal anti-inflammatorydrugs
PVA: Polyvinyl alcohol
